# Non-destructive evaluation method for track slabs damaged by impact of ice blocks detached from high-speed trains

**DOI:** 10.1038/s41598-024-65467-6

**Published:** 2024-06-24

**Authors:** Xiaolin Song, Xuanran Fu, Dingjun Xiao, Cai Yi

**Affiliations:** 1 State Key Laboratory of Rail Transit Vehicle System, Southwest Jiaotong University, Chengdu, 610031 China; 2 School of Environment and Resources, Southwest University Sciences and Technology, Mianyang, 621010 China

**Keywords:** Civil engineering, Characterization and analytical techniques

## Abstract

In winter snowy or rainy weather, the phenomenon of icing under rolling stock during high-speed operation is significantly severe, posing a potential risk of detachment and impact on track structures due to the presence of ice blocks with substantial mass and velocity. Therefore, it is crucial to develop an efficient method for characterizing and evaluating this impact damage in order to assess the service life of the track. To address this issue, the indoor ice impact tests were conducted on track slab models, and a comprehensive analysis was performed on non-destructive testing data before and after the impact test, including 3D surface morphology assessment, surface hardness and wave velocity measurements. Additionally, in order to verify the effectiveness of the nondestructive testing method, the frozen-thawed and not frozen-thawed track slab models were tested and their results were compared. The experimental results revealed that when impacted by ice blocks at a velocity of 100 m/s, small dimples formed on the surface of track slab models with the maximum depth measured at 0.0694 mm. There was a maximum increase rate in surface hardness amounting to 11.61%, and a maximum decrease rate in wave velocity measuring at 6.52%. Furthermore, the impact damage of the two models has been evaluated, the not frozen-thawed track slab model exhibited minor damage after impact, whereas the frozen-thawed track slab model demonstrated moderate damage in the contact region and minor damage outside of that region. The proposed non-destructive testing method effectively enables assessing the impact damage inflicted upon slab models while providing valuable insights for maintenance and repair strategies related to track slabs.

## Introduction

With the rapid and continuous development of Chinese high-speed railways, the operational scope of high-speed rail is becoming extensive, which traverse diverse climatic, temperature zones, multitude of terrains and geological regions. Consequently, the engineering problems arising during the service of rail structures are becoming increasingly region-specific and diverse. The time effect, space effect, and multi-field coupling effect are becoming more pronounced and complicated, which makes it extremely difficult to maintain the stable and safe service of the rail infrastructure in different regions and periods all the times. For instance, the ballastless track service in cold regions is susceptible to multiple types of damages under the complex service environment, thereby directly reducing the safety, stability, and durability of the high-speed railways during its operational lifespan^1,2^.

**Figure 1:**
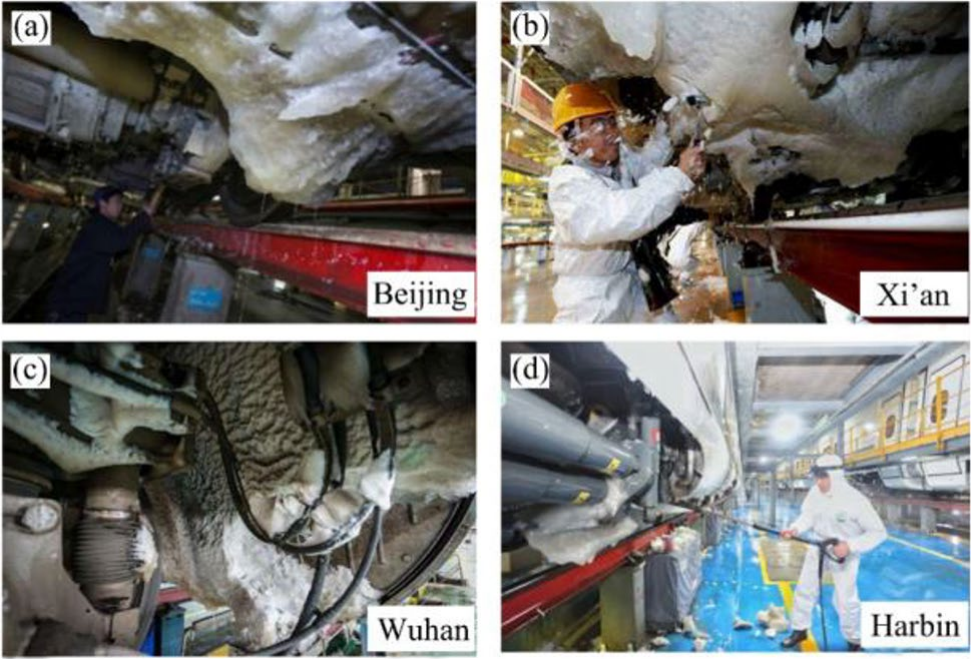
Icing phenomenon on high-speed train bogies in different regions, including (**a**) Beijing. (**b**) Xi’an. (**c**) Wuhan. (**d**) Harbin. (All pictures come from the website).

When a high-speed train runs in winter snowy or rainy conditions, the surrounding rain and snow will move to the outside of the car body due to the formation of negative pressure around the car body^3–9^. Some of the rain and snow adsorbed on the underside of the rolling stock easily freezes into ice. Therefore, to ensure the safety of high-speed trains in ice and snow conditions, measures such as daily ice-melting of vehicles and speed reduction are adopted to reduce the hazards of snow and ice condensation^4–7,10^. Nevertheless, none of these initiatives can eliminate the problem of icing under trains from the root, and the phenomenon of icing of high-speed trains in China is still significantly severe, which is not only limited to high latitude and cold regions but is also very common in central China, such as Xi’an and Zhengzhou^1–4^. Figure 1 from the website shows the icing under rolling stock after a moderate snowfall in China in 2018. With the increased vibration of the vehicle and the change in external temperature, some of the ice attached to the underside of the trains will fall off without warning. The ice block detached from a high-speed train will impact the track and other infrastructure at a very high speed, which may cause deformation and damage to the track slab, thereby reducing its service life. As the continuous expansion of the national high-speed railway network, the impact damage problem of falling ice on the track will become more and more serious. Therefore, it is urgent to study the deformation damage of the track under the impact of ice, thereby providing valuable insights for maintenance and repair strategies related to track slabs.

The understanding of the interaction and impact failure mechanisms between ice and structures has made significant progress in recent years. Most research efforts have been focused on investigating the high-speed impacts of ice balls on composite plates^11–17^ and the effects of moving ice on structures^18–23^. These research endeavors have primarily employed experimental methods and numerical simulations. In the context of ice block impact on composite panels, current research employs various methodologies, including pure experimental investigations and a combination of experiments and numerical simulations. In terms of experiments, the research focuses on several aspects, encompassing the characterization of ice block impact damage, the influence of ice block geometry on damage to composite panels, and the nature of crack damage in composite panels, among others^11–13^. In the realm of numerical simulation, researchers have employed software like Abaqus^24^ to simulate a wide range of materials, including composite panels, Taylor bars, and CFRP subjected to ice impact. For instance, Tippmann et al. developed strain rate-dependent ice projectile models using Abaqus software. They conducted both experimental tests and corresponding simulations of high-speed ice projectile impacts on Taylor bars^14^. The accuracy of these models has been validated through experimental testing, enabling investigation into deformation, delamination damage, laminate crack propagation, and damage patterns in composite laminates under ice impacts^14–17^. The aforementioned studies exclusively focus on the impact of ice balls on composite panels, while in terms of the influence of floating ice on structures, scholars have made significant progress in elucidating the dynamic interaction between ice and structure. Splitting failure is an important failure mode when ice interacts with structures, which has been observed and studied by using unconstrained rectangular floating ice with different sizes and indentation velocities in different literature^22,23^. Furthermore, Li et al. developed refined finite element models to simulate the process of drift ice impacting bridge piers, enabling them to collect vital data on ice impact forces under various ice raft characteristics^18^. Timco et al. compiled datasets related to the impact of floating ice on structures and explored the correlation between impact forces and the kinetic energy of drifting ice during impact^19^.

In summary, the impact of ice blocks leads to varying degrees of damage in both metal plates and concrete structures. However, detecting damage in concrete can be more challenging compared to metal martials due to its complex composite nature, rendering some traditional testing techniques less suitable. Consequently, the detection of concrete damage has emerged as a significant research field. Currently, various non-destructive testing (NDT) methods are available for concrete evaluation, including the Coin tap test, Acoustic Emission (AE), Impact Echo testing, ground-penetrating radar, Digital Image Correlation (DIC), and ultrasonic pulse velocity^25–31^. The Coin tap test, as a simple pulse-echo method, represents one of the oldest NDT techniques. The AE method, on the other hand, offers unique advantages for monitoring concrete damage. For example, it can detect the early stages of corrosion in reinforced concrete, providing a promising approach for quantitatively assessing the corrosion process resulting from the expansion of corrosion products^32–34^. The Ultrasonic Pulse Velocity (UPV) method, another well-established NDT approach, assesses the relative condition of concrete by measuring the duration of ultrasonic pulses over a known path length. In recent times, the high-precision DIC technique has enabled highly accurate full-field strain observations of concrete. Zhou et al.^35,36^, for instance, employed a combination of the DIC technique and wavelet packet analysis to achieve more accurate localization of microscopic concrete damage locations. Similarly, Zhao et al. used the same method to predict the emergence of microscopic cracks in concrete, effectively reflecting the damaged state of concrete under various loading conditions^25^.

In a word, in the studies of hail impacting aircraft and composite plates, the ice mass is light and the relative impact velocity is high, while it has a large mass with a low velocity in the term of sea ice and river ice impacting ships, piers, and dams. It is easy to find that the ice blocks involved in these two types of problems vary significantly in both mass and velocity, differing by several orders of magnitude. However, the mass and impact velocity of the detached ice from high-speed trains falls within the intermediate range, which makes it difficult to directly apply the aforementioned research results to this issue. Therefore, this paper proposes an efficient and simple method to non-destructively evaluate the ice impact damage of the track slab, including assessment of 3D surface morphology, measurement of surface hardness, and wave velocity measurements. Although these methods are known, they are here applied to solve a new problem in rail structure.

## Impact tests steps and non-destructive testing methods

**Figure 2:**
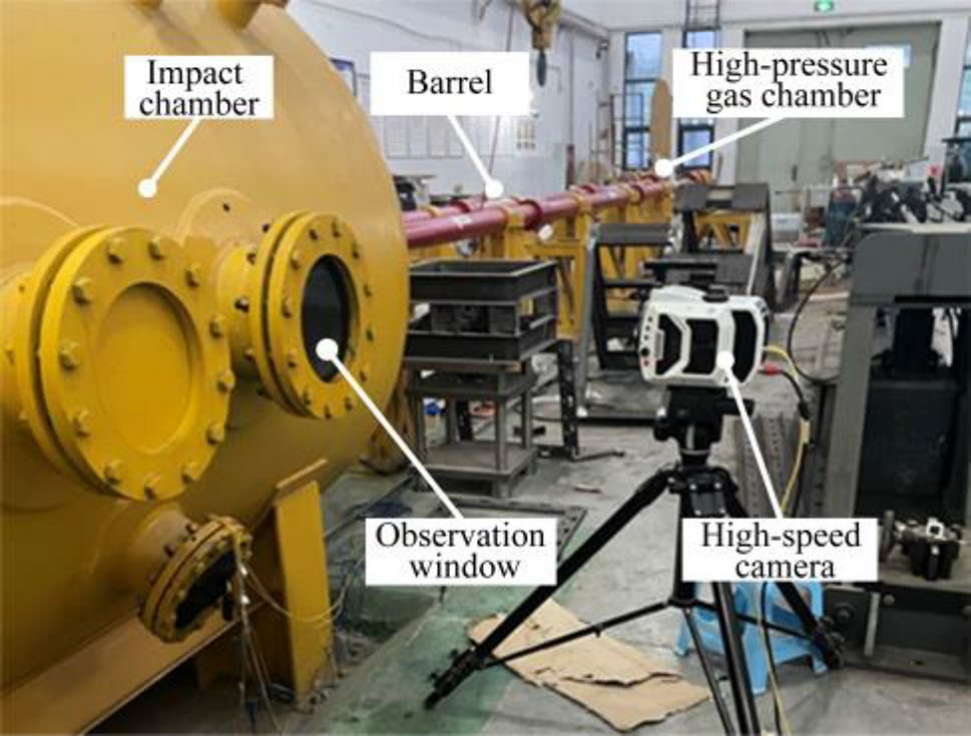
On-site photo of the impact test system.

Since the time and location of ice detachment from high-speed trains are very random, it is difficult to detect the impact position and evaluate the damage of the track slab through field tests. Therefore, in this paper, a simplified indoor test is conducted to achieve this issue. Specifically, a one-stage light gas gun is used to project the prefabricated ice onto the track slab model. Before and after the impact experiment, the 3D surface morphology, surface hardness, and the ultrasonic wave velocity of the track model are all measured and compared, through which the surface deformation and impact damage characteristics can be evaluated from the changes of the three measured values. Observations are also made by high-speed video during the impact event and the crushing of the ice block can be recorded (see Fig. 2).

### Light gas gun equipment

**Figure 3:**
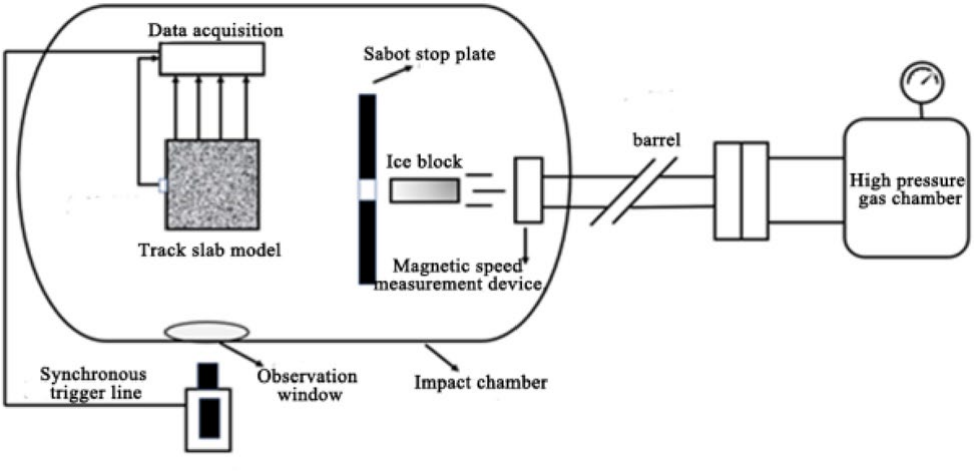
Schematic diagram of light gas gun equipment.

The impact test was carried out on a one-stage light air cannon (inner barrel diameter 76 mm) in the laboratory of blasting and impact dynamics at Southwest University of Science and Technology (SWUST), China. The high-velocity impact system (see Fig. 3) consists of multiple pieces of equipment: light gas gun, sabot stop plate, magnetic velocimetry system, and impact test chamber.

The breech-loading light gas gun is 12 m long and has two possible barrel inner-diameter sizes, 37 mm, and 76 mm. Projectile weight ranges from 0.2 g to 4 kg with a velocity varying from 40 to 2000 m/s.

**Figure 4:**
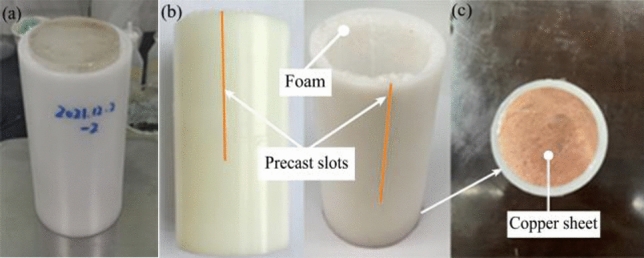
Images of sabot. (**a**) Ice block placed in the sabot. (**b**) Precast slots of the sabot. (**c**) Copper sheet pasted on the sabot tail.

To protect the ice block and the barrel lining of the light gas gun, the sabots were required to project the ice through the barrel without damage. The consistent manufacture of ice blocks and sabots is very important for the repeatability of experimental results. The sabot is a cylindrical drum with an outside diameter of 76 mm, slightly less than the diameter of the light gas gun bore. It is constructed of low-density expanding rigid polyurethane (PE) foam with four preformed slots (see Fig. 4a,b). A thin layer of foam placed between the ice and the sabot shell can not only prevent the ice from melting too quickly but also play a role in fixing and cushioning.

**Figure 5:**
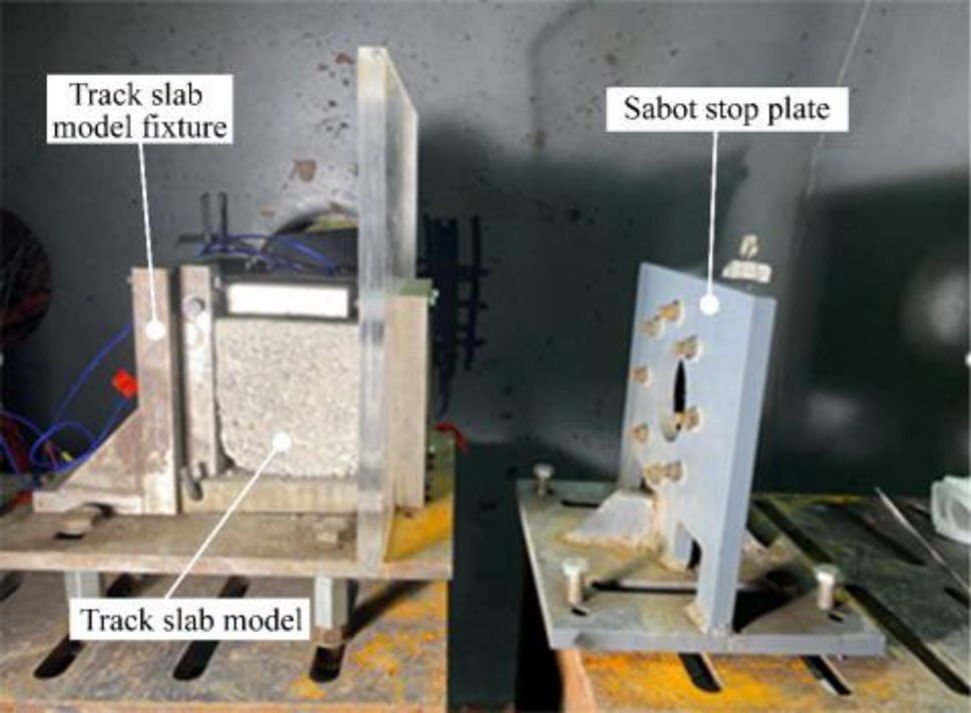
Sabot stop plate and track slab model.

The sabot stop plate is a 15 cm thick steel plate with a 70 mm diameter hole as shown in Fig. 5, which is large enough to accommodate a projectile and small enough to accommodate the sabot. When the ice block packaged by the sabot is transported to the sabot stop plate, the splitting sabot is stopped by the plate and the ice projectile is released cleanly and smoothly at the pre-set speed. The velocity is measured by a magnetic velocity system located in the projectile path immediately after the sabot stop plate. A round copper sheet of 0.1 mm thickness is pasted onto the tail of the sabot as shown in Fig. 4c, as the projectile passes through the magnetic velocimetry system, the velocity is determined according to the change in magnetic flux in the magnetic velocimetry device. The impact velocity of the ice block can be controlled by adjusting the air pressure of the light gas gun.

As the impact force of an ice block at a very high speed is very powerful, a special fixture, shown in Fig. 5, is made to hold the test model against movement and rotation. The fixture is placed on the test bench, which is fixed in the chamber by the bolted connection. A Plexiglas baffle is tightly fixed in front of the test model to prevent a large number of small ice fragments from spraying and hiding the model from the video camera.

**Figure 6:**
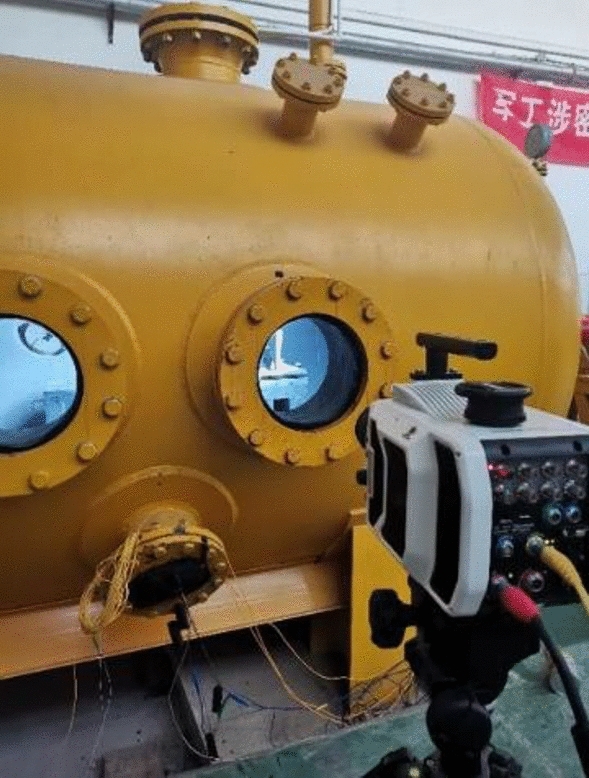
High-speed camera positioned outside impact test chamber.

The impact tests are visually observed by a high-speed video camera (Phantom V2012 CMOS) with a pixel size of 28 microns and a bit depth of 12 bits, positioned outside the viewing window of the impact test chamber (as shown in Fig. 6). Its frame rate is approximately 89,000 frames/s with a shutter speed of 10 μs to record the images of the impact process. The video is instrumented to directly observe the impact progress of the ice block and the separation of the ice from the sabot, which directly determines the validity of the impact test. It is essential for the ice to remain intact and undamaged when it leaves the sabot and reaches the target.

### Ice projectile

Since it is hard to obtain the actual ice blocks from high-speed trains and keeping them in good condition for testing, ice projectiles are produced in the laboratory. It is true that the physical properties of the ice samples we made may be different from the actual ices, but this difference does not exert decisive influence on the form of damage to the track slab.

According to the study, the ice block detached from a high-speed train is irregular in shape and varies greatly in weight, with the maximum weight exceeding 2 kg. Based on the results in reference^24^, a cylindrical impactor causes greater residual deformation than a conical or inclined impactor, so the shape of the experimental ice is chosen as a cylinder. In order to maximize the mass of the ice block, its diameter is set at 60 mm based on the inner diameter of the barrel of the light gas gun used. The length of the ice block is set at 140 mm according to the recommended length/diameter ratio of 2.33 given in reference^37^. The ice block has a mass of approximately 340 g and a density of 896 kg/m^3^.

**Figure 7:**
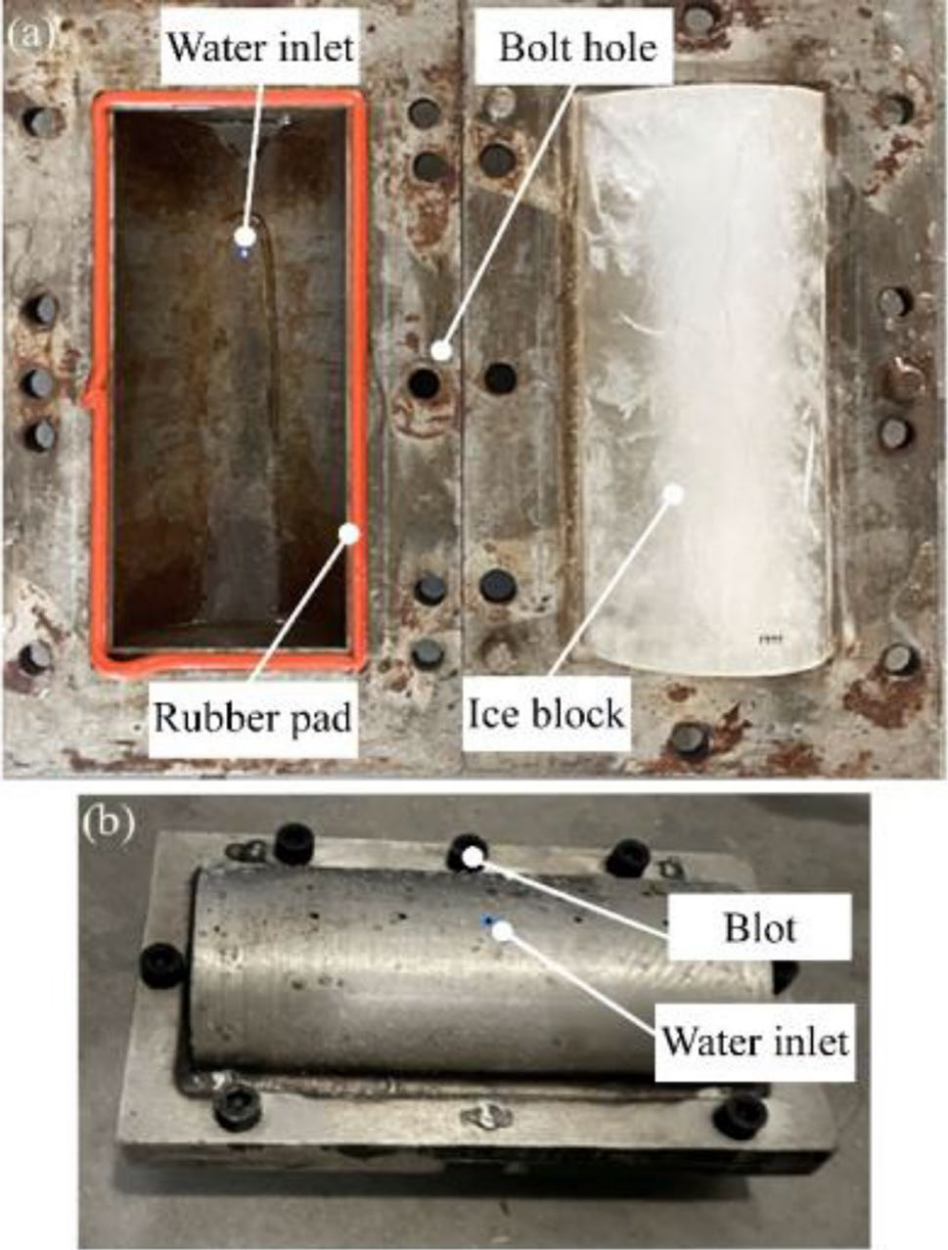
Ice-making mould. (**a**) Mould in the open position. (**b**) Closed mould.

The ice-making mould, shown in Fig. 7, is made of steel because of the high frost-heaving force. It is divided into upper and lower parts for ease of demoulding and is bolted together. A rubber pad is installed at the joint of the mould to ensure the sealing effect. A water injection hole is located on the top of the mould, from which the water can be filled and overflows freely during the freezing process. Discs can be placed to change the length of the ice blocks, and the length and nose shape of the ice blocks can be conveniently changed by embedding different shaped modules in the mould.

As the naturally frozen ice on the high-speed train usually has impurities and air bubbles inside, the ice used in the test is made with tap water instead of distilled or deionized water. The mould with the water is placed in a freezer at − 18 °C (± 2 °C) for at least 48 h. The ice projectile is removed from the mould and immediately placed in the freezer with a sealed plastic bag.

Due to the primary focus of this study on non-destructive testing methods for track slab damage under ice block impact loads, the characteristics of the ice block, which serves as the impactor in this experiment, are not central to this paper. Therefore, in this paper, only compressive strength of the ice block was provided in this paper. The investigation of other mechanical property parameters, such as Poisson's ratio, modulus of elasticity, and yield strength for ice blocks, will be systematically explored in subsequent research.

**Figure 8:**
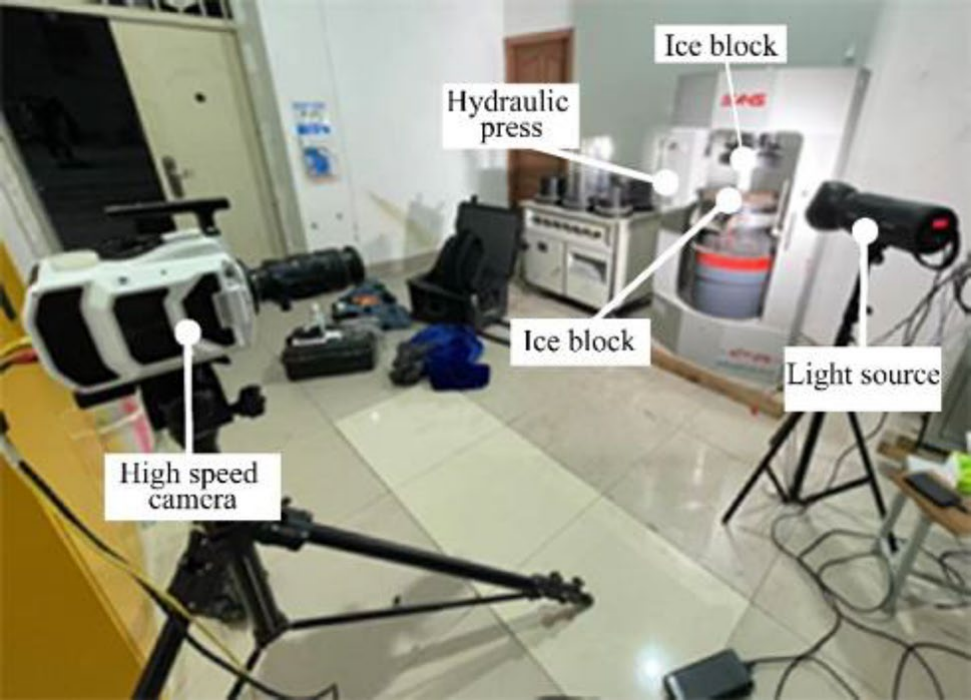
Compressive strength tests of ice blocks.

**Figure 9:**
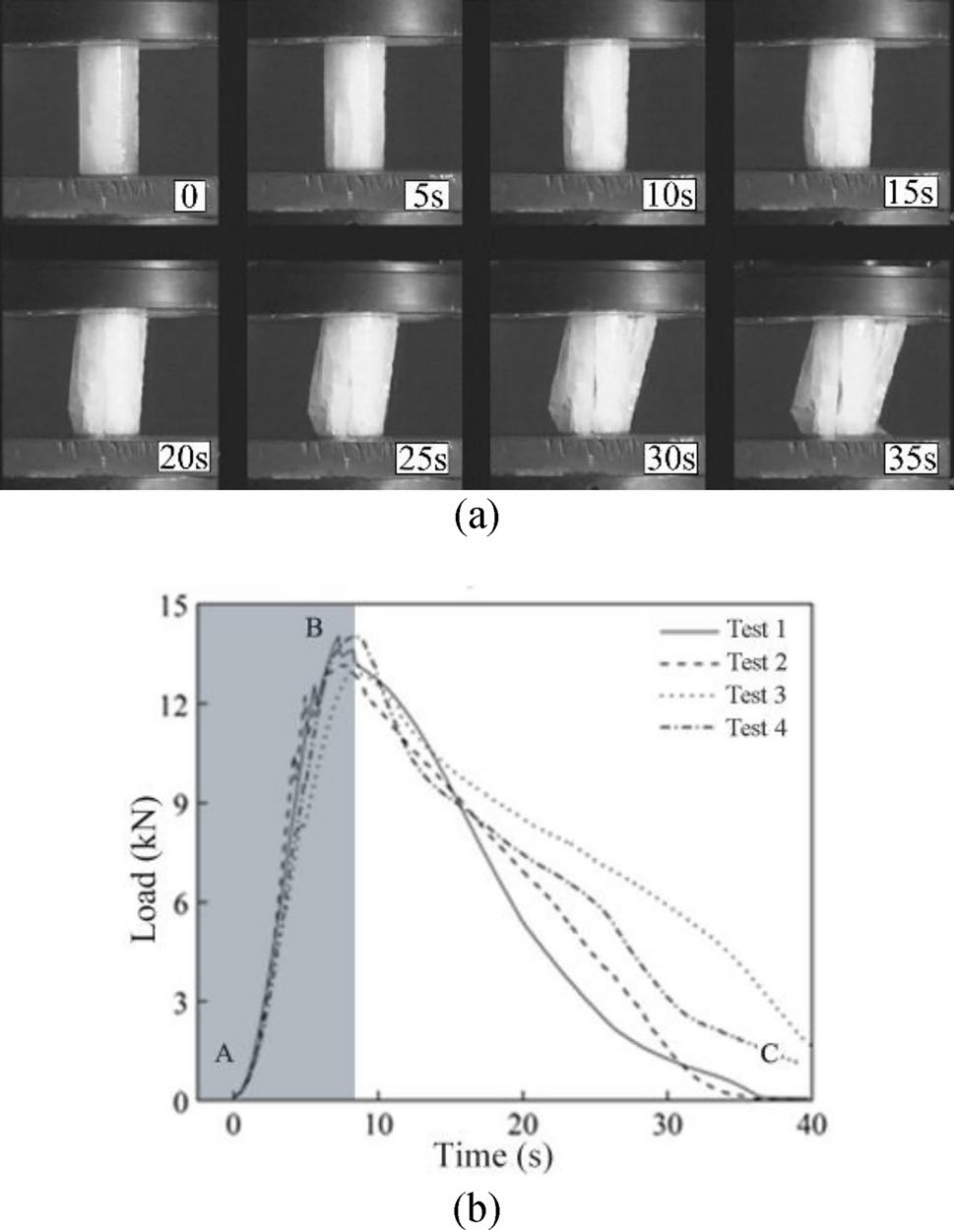
Compressive strength test results of ice blocks. (**a**) Compression failure process. (**b**) Load-time curves.

The compressive strength tests of the laboratory-manufactured ice blocks were carried out in winter with a temperature range from − 2 to 5 ℃, as illustrated in Fig. 8. The compression failure process and the load curves of the 4 samples are shown in Fig. 9. It is evident that the A-B segment is the linear elastic stage, where the ice block is compressed and longitudinal cracks start to form inside; the B-C segment is the fracture stage, where the longitudinal cracks inside the ice block gradually penetrate, and the load reaches the limit at point B, the ice block begins to break, but does not directly lose its compressive strength, instead, the ice begins to slip, slowly swell and crack until they collapse and slide off and gradually lose their compressive strength. The four curves show good repetitiveness, which indicates the test and instruments are valid and credible.

By utilizing ultimate load value at point B in Fig. 9b, the compressive strength of the ice block can be calculated. The experimental results suggest that the average value of the compressive strength of the ice projectile is about 4.70 MPa, and its maximum difference under each test is only 7.5%.

### Manufacturing of track slab model

As the size of the ballastless track, a multi-layered structure, in the Chinese high-speed railway is too big (for instance, CRTS III ballastless track slab with a length of 5600 mm, width of 2500 mm, and thickness of 210 mm) to be arranged in the impact test chamber, it is not possible to use the real slab track for the impact test directly. Therefore, simplification of the ballastless track structure becomes necessary.

Before conducting the impact test, a full-scale model of the CRTS III ballastless track slab was constructed to simulate the effects of ice impact on the track structure using Ls-dyna software. The simulation results indicate that most of the impact damage occurs in the track slab layer, while damage to the self-compacting layer and base plate is minimal and can be considered negligible. Additionally, the range of impact damage falls within a square area measuring 150 mm × 150 mm^38^. Based on this finding, the multi-layer ballastless track structure can be simplified as a 150 mm × 150 mm × 150 mm cube structure. Simulation results demonstrate that there is little disparity in terms of damage and dynamic response between the simplified track slab model and its full-size counterpart^39^. Therefore, for ease of manufacturing, we propose simplifying the ballastless track structure as a prefabricated concrete cube measuring 150 mm squared, which serves as standard specimen for material parameter measurement.

Although our simulation results have demonstrated that impact damage occurs within the track slab layer itself, it is essential to acknowledge that the actual ballastless track structure functions as an integrated system comprising various components, material interfaces, and structural interactions that can influence the propagation and distribution of impact forces and damage mechanisms. Factors such as variations in material properties, interfacial bonding, and load transfer mechanisms between layers may potentially affect the patterns and severity of damage. However, this study aims to propose and validate a non-destructive testing method for evaluating ice impact damage; therefore, these aforementioned factors are not considered in this paper. Nevertheless, it should be noted that these factors also serve as limitations of the simplified model used in this study and requiring further improvement in future research.

The track slab model with a strength grade of C40 consists of P.O. 42.5 grade ordinary Portland cement, coarse aggregate (gravel with a particle size of 5–10 mm), fine aggregate (common river sand), and polycarboxylic acid high-performance water-reducing agent. All the gravel is filtered several times through a 10 mm aperture sieve and used after drying to prevent the sediment and water contained in the gravel from affecting the strength of the model. The selected proportion of the concrete mix is listed in Table 1.

**Table 1:** Concrete mix proportion of the model.

Strength grade	Cement	Gravel	Sand	Water	Water-reducing agent
C40	1	2.31	1.67	0.45	0.0038

The mixed concrete is poured into the moulds placed on the vibrating table and vibrated until it is fully compacted. The prepared model can be demoulded after standing for at least 24 h. Only after a 28-day standard curing, can the model be used for experiments.

**Figure 10:**
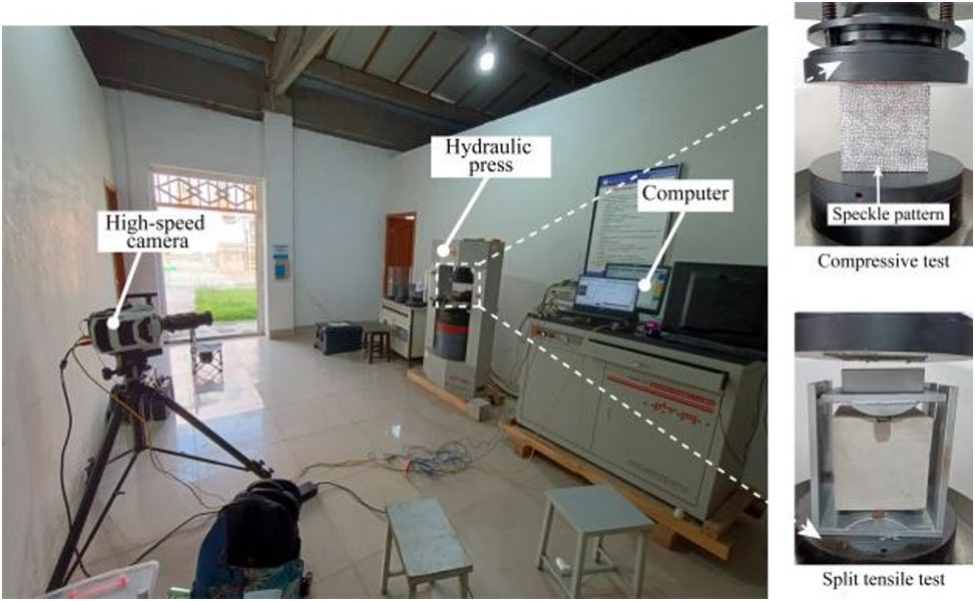
Compressive and tensile strength test of track slab model.

Subsequently, the compressive strength test and split tensile test are conducted on track slab specimens from the same batch, following the guidelines specified in the Standard for Test Methods of Physical and Mechanical Properties of Concrete (GB/T50081-2019)^40^. The schematic depiction of two kinds of tests is presented in Fig. 10.

**Figure 11:**
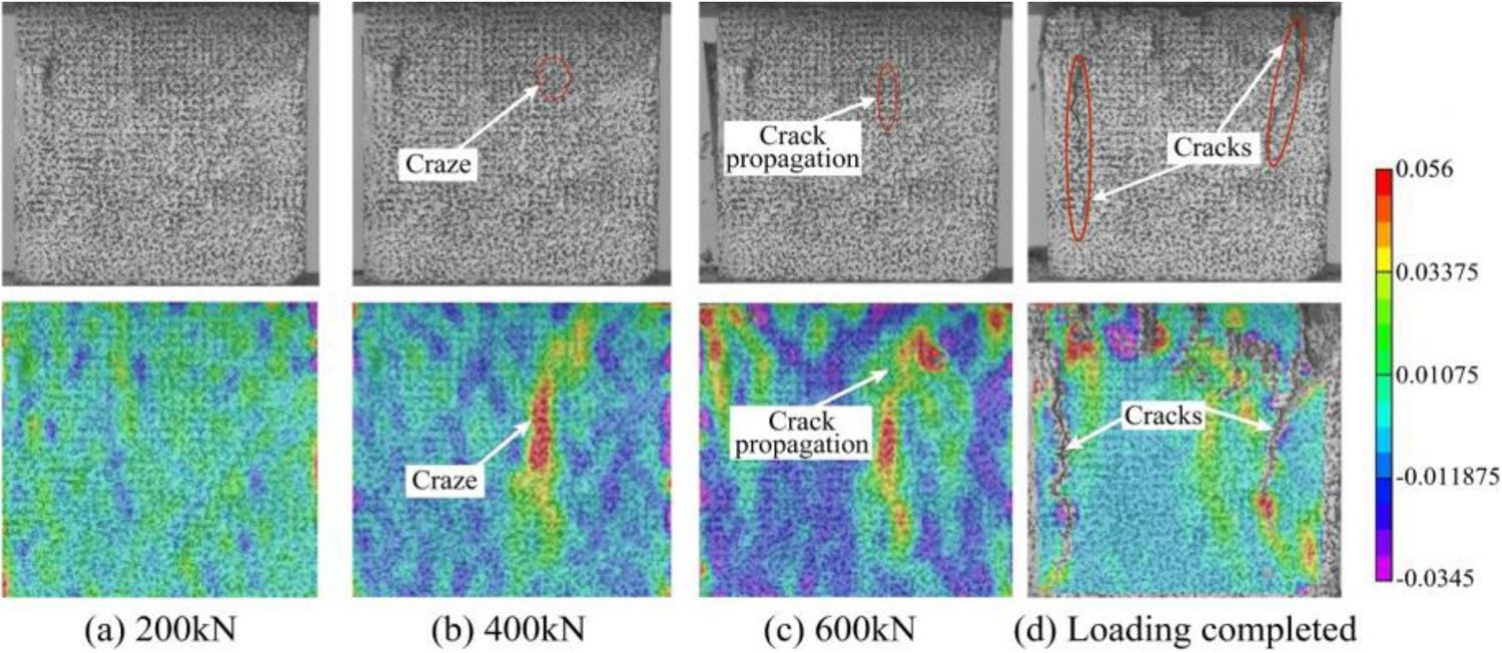
Compression failure pattern (above) and DIC stress contour (below) of track slab under different loads.

**Figure 12:**
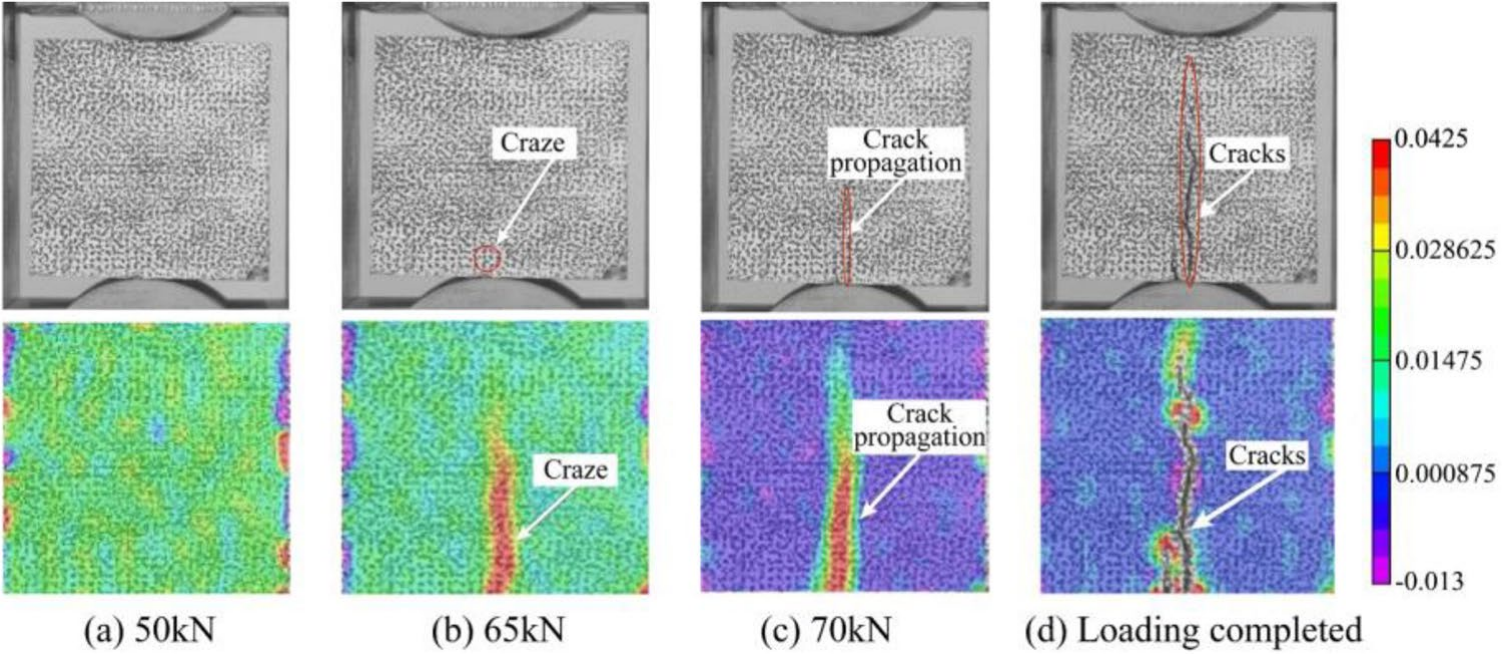
Tensile failure pattern (above) and DIC stress contour (below) of track slab under different loads.

The YAW4106 microcomputer-controlled constant loading pressure tester of the electric type was employed for this purpose. Throughout the testing procedure, the high-speed camera is utilized to capture the failure process of the track slab model (Figs. 11, 12, top row), while employing the Digital Image Correlation (DIC) technique to calculate strain measurements during this process (Figs. 11, 12, bottom row).

Notably, it can be observed that areas exhibiting elevated tensile and compressive stress within the strain field correspond remarkably well with the locations of crack formation. The DIC technique demonstrates a noteworthy capability in accurately predicting the onset of crack formation as well as the direction of their propagation. The results of the compressive and tensile tests are presented in Table 2. These results indicate that this batch of track slab model meet the strength grade of C40.

**Table 2:** Compressive strength and tensile strength of track slab model.

Test number	Compressive strength/MPa	Tensile strength/MPa
1	42.62	4.17
2	42.11	4.10
3	41.82	4.09
Average	42.18	4.12

### Nondestructive testing methods

Non-destructive testing (NDT) provides essential method data to determine if damage has occurred inside the slab model without destroying the specimen. There are more and more NDT techniques, and each NDT technique has specific benefits and limitations. By combining multiple NDT analysis methods, a more comprehensive characterization and evaluation of damage is possible. There are three general NDT methods employed in this paper, which are 3D surface morphology scanning, surface hardness measurement, and ultrasonic wave velocity measurement. Through comparison of the variation of the three measured values before and after the impact test, a complete characterization and evaluation of the damage for the slab model is presented.

#### 3D surface morphology scanning

**Figure 13:**
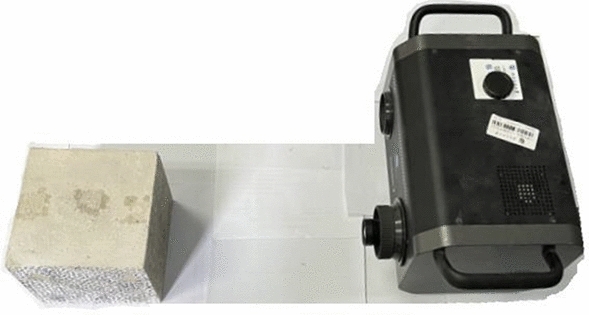
3D laser scanner.

The 3D topography surface morphology scanning is accomplished by the 3D laser scanner from Konica Minolta Range7, with a resolution of 1280 × 1024 pixels, an accuracy of ± 40 μm in the scanning plane and ± 4 μm in scanning depth direction, as shown in Fig. 13.

**Figure 14:**
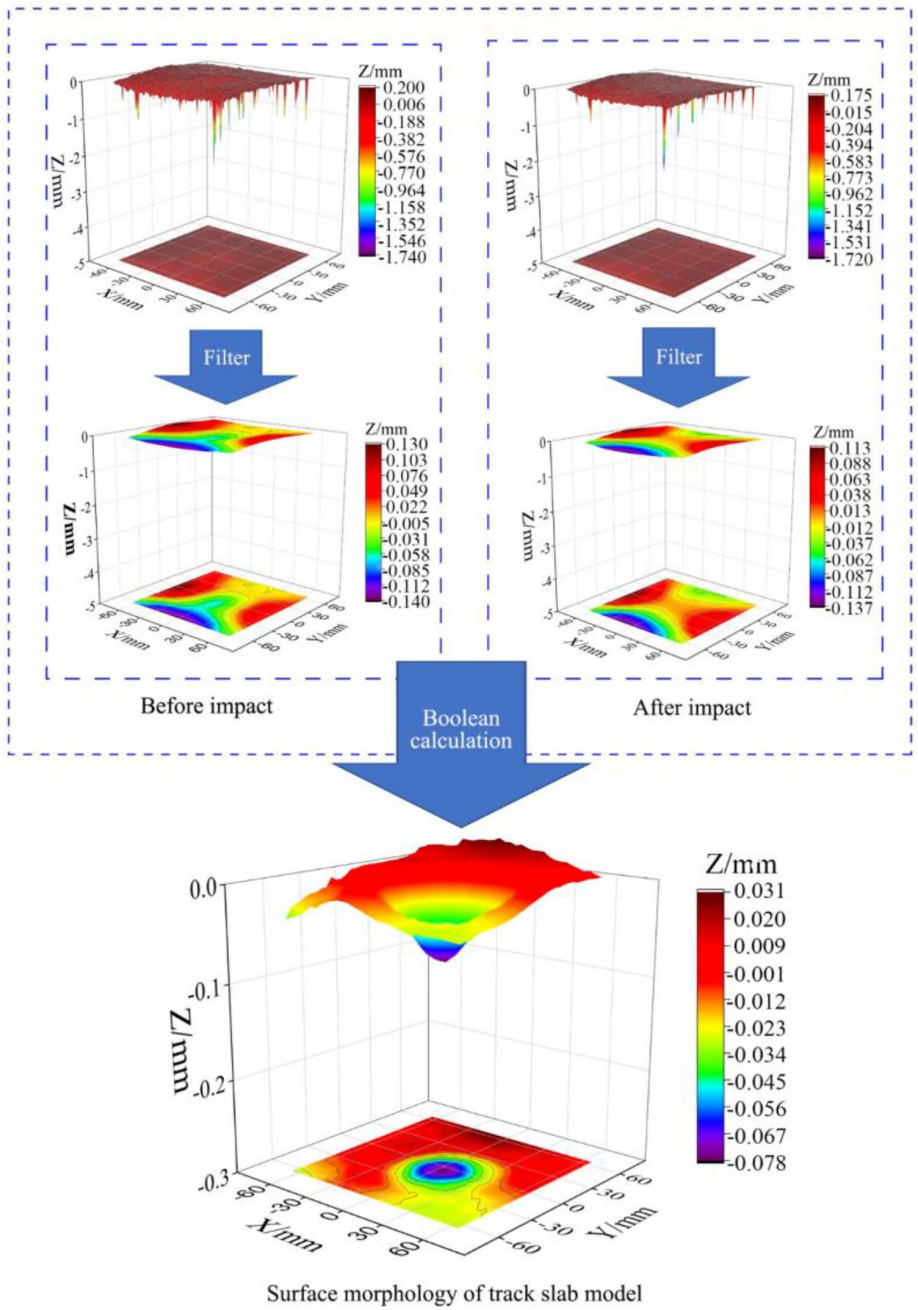
Surface morphology procedure from point cloud data.

The scanned original point cloud data can be processed with the Cloud-Compare software delivered with the device or other business ones. The 3D surface morphology of the slab model excited by impact can be Boolean calculated on the point cloud data before and after the impact test. The specific processing steps are shown in Fig. 14.

#### Surface hardness measuring

**Figure 15:**
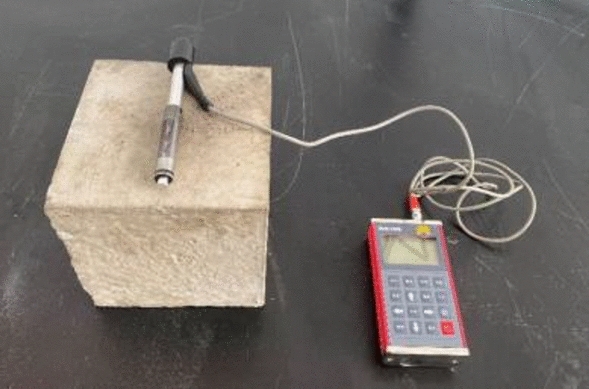
RH100 high precision hardness measuring instrument.

In order to grasp the variation of the surface hardness under the impact load, the surface hardness is measured by the RH100 high precision hardness measuring instrument produced by Beijing Times Ruiguang Technology, as shown in Fig. 15.

**Figure 16:**
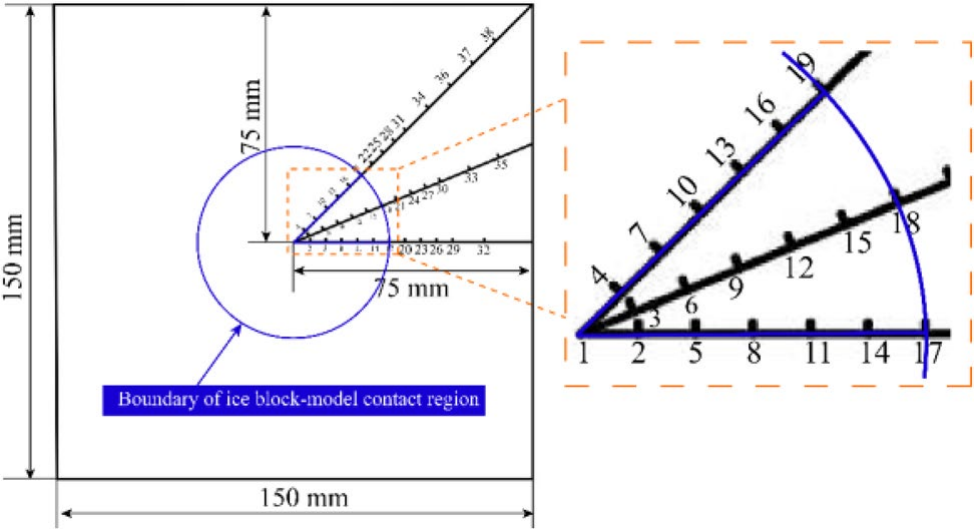
Diagram of measurement points for hardness and wave velocity.

In accordance with the symmetry of the projectile and the target, for ease of measurement, the hardness measurement points are distributed along three lines starting from the contact region between the ice block and the track slab model (in the subsequent text, this region will be referred to as the “contact region”), as shown in Fig. 16, the contact region is at measuring points 1–19. The angle between the three lines is 22.5°. The spacing of the measurement points in each line is 5 mm within 50 mm of the centre, outside the region the spacing is 10 mm.

The hardness is measured five times randomly selected near each measurement point, if no evident anomalies are observed in these measurements, the average of the five values can be employed as the hardness value for this measurement point. However, in case any outliers emerge, it is necessary to re-measure and re-calculate the average hardness value for that particular measurement point.

#### Ultrasonic wave velocity measuring

**Figure 17:**
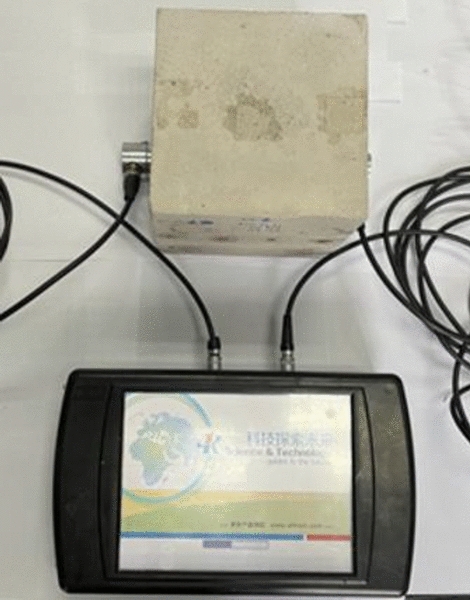
RSM-SY5 wave speed measuring instrument.

The ultrasonic wave velocity is measured by the RSM-SY5 wave speed measuring instrument (as shown in Fig. 17) developed by Wuhan Zhongyan Science and Technology Co., Ltd. The distribution of the model ultrasonic wave velocity measurement points is consistent with that of the hardness test point, while the average ultrasonic wave velocity is calculated by three measuring points, each of which has no outliers. The coupling agent Vaseline is evenly applied on the surface of the ultrasonic probe to maintain a tight fit with the surface of the model.

## Results and discussions

In order to better demonstrate the effectiveness of non-destructive testing method, two control groups with freeze–thaw cycles of 0 and 100 were selected in this paper, each control group consisted of nine track slab models: three for compressive testing, three for tensile testing, and three for pre- and post-impact non-destructive testing. In addition, a track slab model with better regularity of NDT results from each of the two control groups was selected for comparative analysis. The mechanical properties of the prefabricated track slab model and the ice projectile from the same batch with the impact test were first obtained by the standard tests. Then, the 3D surface morphology visual inspection, surface hardness, and ultrasonic wave velocity measurement were carried out on the same specimen to perform the impact test.

Due to the height between the ballastless track support bed and the bottom of the high-speed train is relatively small, when ice blocks detach from the high-speed train, the vertical velocity component can be regarded as negligible. Thus, the ice block will possibly vertically impact the side of the track support bed with same velocity of the high-speed train at detachment. Furthermore, this study considers worst-case scenarios: ice block vertically impacts the track slab model at an equivalent speed of a high-speed train to investigate their effects and validate our non-destructive testing effectiveness. Hence, for this study, an approximate impact velocity of 100 m/s has been selected to vertically impact the track slab model.

The ice projectile was loaded by the light gas gun and impacted the track slab model at the speed of 100 m/s within 3 min. After the impact test, the same NDT was carried out to assess the damage caused by the impact. This procedure was repeated for each impact. The impact test and measurements were performed at least twice for each test condition to ensure the accuracy of the test. The results of the impact test and measurements at an impact are presented below.

### Validation of the efficacy of the impact test

**Figure 18:**
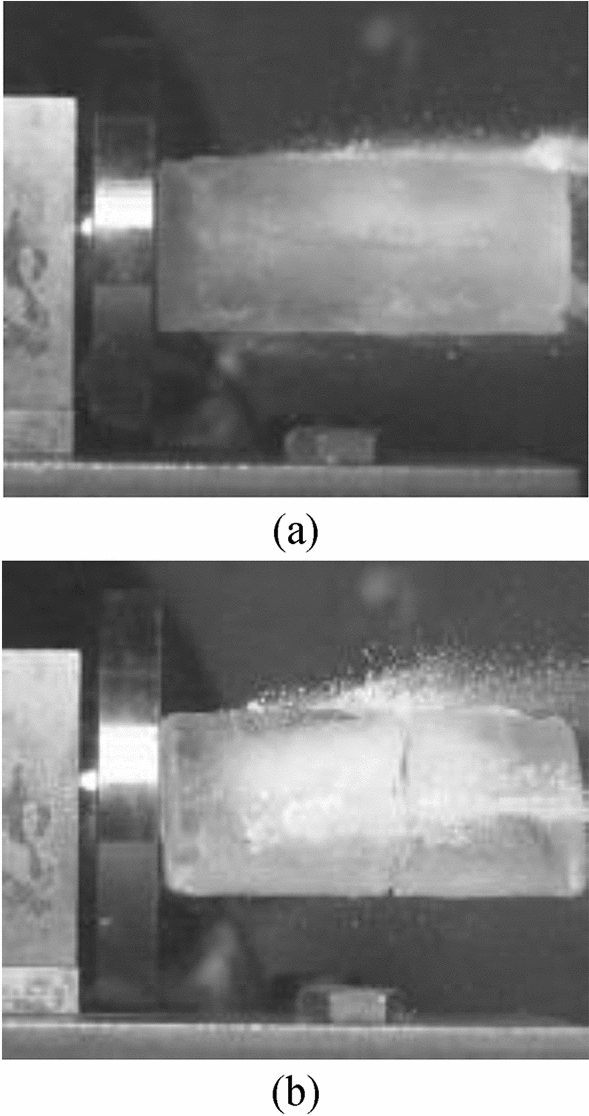
Integrity of ice block. (**a**) Intact ice. (**b**) Fractured ice.

**Figure 19:**
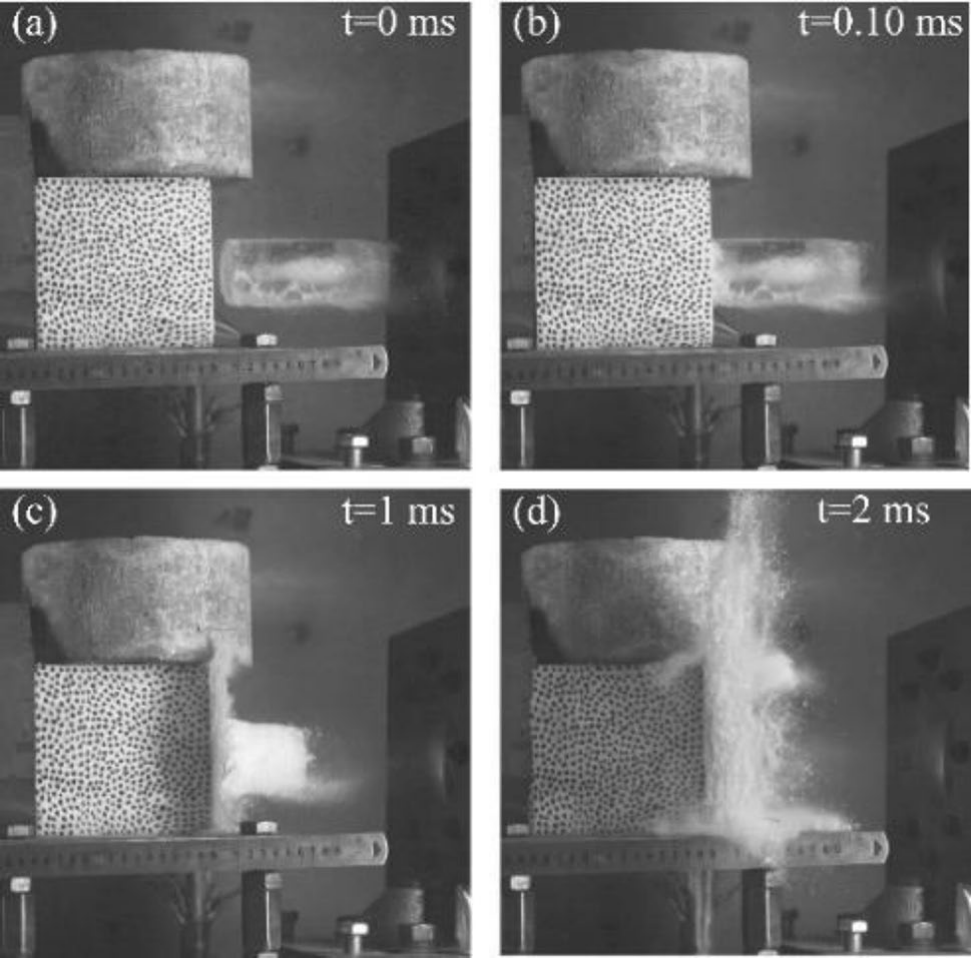
Impacting fragmentation process of ice block at different times.

The Ref.^36^ points out that the amplitude of the impact force will be significantly diminished if the ice breaks before contact with the target, which will influence on the precision of the impact test. Therefore, all experiments must ensure the ice blocks remain intact before impact (as shown in Fig. 18a). If the ice blocks are fractured (as shown in Fig. 18b), the experiments should be deemed invalid. Hence, to ensure experimental efficacy, this study employed a high-speed camera to record the process of the ice block impacting the track slab model, ensuring that the shape of all ice blocks involved in the experiment remained intact after they deviated from the stop plate. The images that depict the impacting fragmentation process of ice block at different times are shown in Fig. 19.

At the moment of impact, *t* = 0 ms, the ice block is on the verge of contacting the track slab model. Although the ice block has some air bubbles inside it, the ice block maintains its structural integrity without discernible cracks and exhibits nearly transparency, as illustrated in Fig. 19a. In Fig. 19b, it can be observed that upon the ice block impacts the track slab model, it is compressed by the track slab model, resulting in localized fragmentation and the emergence of fragments. Notably, the fractured section of the ice block assumes a white appearance, while the unfractured portion retains its transparency. Subsequently, as the ice block continues to impact the track slab, at *t* = 1.0 ms, cracks propagate throughout the entire ice block, causing the entire block to appear white, as depicted in Fig. 19c. By *t* = 2.0 ms, the ice block experiences catastrophic fragmentation, as depicted in Fig. 19d, and the ice block essentially disappears. The complete observation of the ice impact process on a track slab model substantiates the validity of the impact test.

### Impact damage evaluation based on nondestructive testing

**Figure 20:**
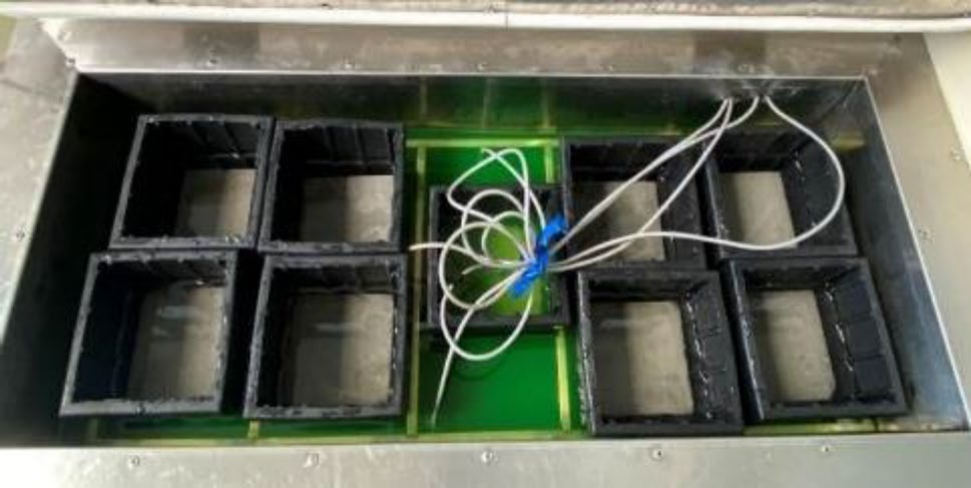
Track slab models in the freeze–thaw box.

The damage of the track slab model was assessed using non-destructive testing data, which included 3D surface morphology, surface hardness, and wave velocity measurements taken before and after the impact test. Additionally, the track slab model subjected to 100 freeze–thaw cycles was also analyzed, as shown in Fig. 20. The comparison between the frozen-thawed model and the not frozen-thawed model was conducted to validate the effectiveness of the non-destructive testing method.

It should be pointed out here that the track slab model undergoing freeze–thaw cycles has completely thawed when impacted. Because, in the frozen model, there will be ice in the internal pores and cracks, which will fill the original voids and cracks, and may increase the strength of the model. To investigate the damage impacted by ice block under worst-case scenarios, a completely thawed track slab model was selected for experimental analysis.

#### 3D surface morphology of track slab model

**Figure 21:**
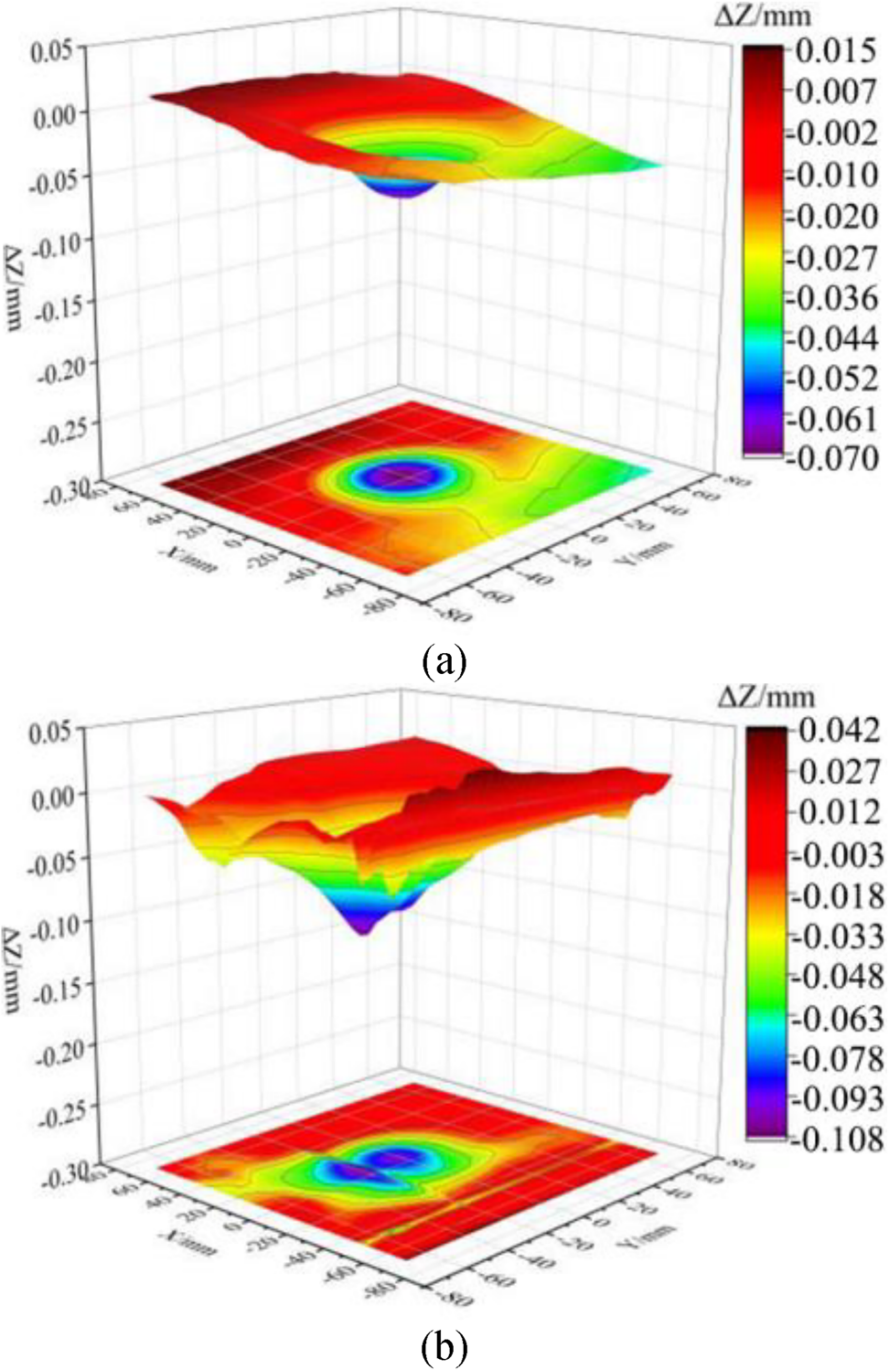
Three-dimensional surface morphology of the track slab model after impact. (**a**) Not frozen-thawed model. (**b**) Frozen-thawed model.

Figure 21 depicts the three-dimensional surfaces of two track slabs after being impacted by cylindrical ice blocks with a diameter of 60 mm with a speed of 100 m/s. It can be observed that both track slab models have developed a small dimple with a diameter close to the ice block on their surfaces. The maximum depths of these dimples are located near the center of impact, measuring 0.0694 mm and 0.108 mm for the not frozen track slab model (shown in Fig. 21a) and the model subjected to 100 freeze–thaw cycles (shown in Fig. 21b), respectively.

Due to the track slab model primarily consisting of concrete, the water in the pores and cracks of the concrete expands and contracts during freeze–thaw cycles, creating internal stresses and strains in the model. The repeated expansion and contraction of the water weakens the cohesion and internal friction of the track slab model, resulting in the reduction of its strength and stiffness. The reduced strength of the concrete makes it more susceptible to deformation and settlement under the ice impact load, leading to the increase of the depression depths of the track slab model. Therefore, it is indicated that the track slab model subjected to 100 freeze–thaw cycles exhibits significantly greater depression depths compared to the not frozen one, but both depressions have diameters roughly equivalent to the ice block diameter. Therefore, the utilization of 3D surface scanning technology not only allows for the timely localization of damage on the impact surface but also enables the quantitative characterization of such minor damages.

#### Hardness results of the track slabs impact surface

**Figure 22:**
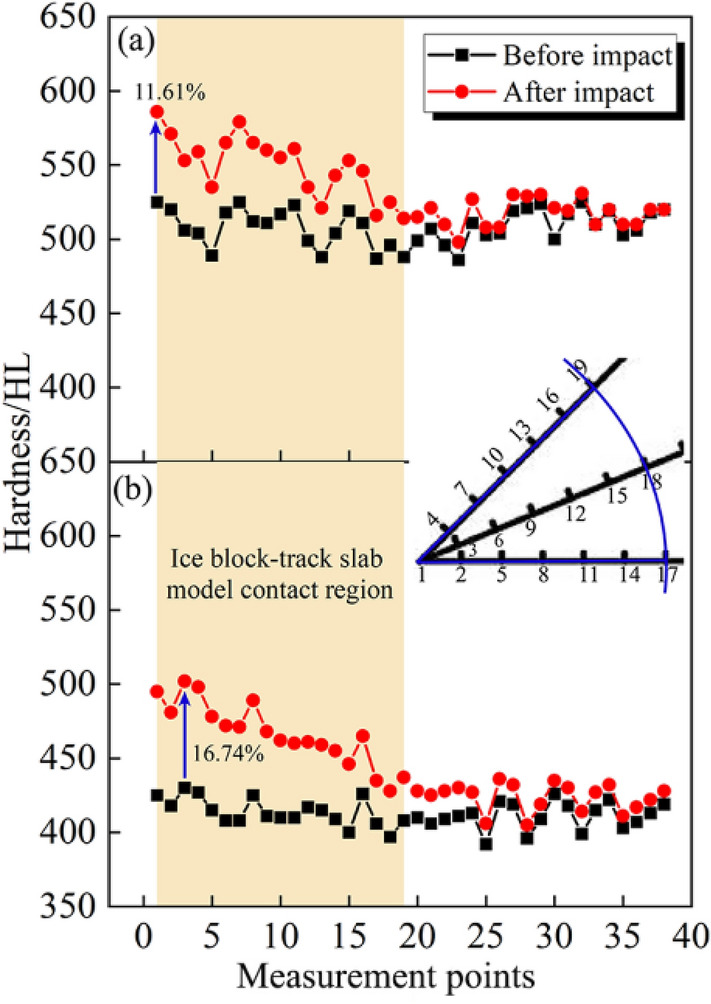
Surface hardness comparison before and after impact. (**a**) Not frozen-thawed model. (**b**) Frozen-thawed model.

The measurement data of surface hardness before and after impact are displayed in Fig. 22.

As shown in Fig. 22a, after the impact of the ice block, the surface hardness will increase obviously in the contact region, while the increment becomes less noticeable as measurement points far away from the contact region. Specifically, at measurement point 1, the surface hardness of the model exhibits the highest growth rate, which is 11.61%. Furthermore, within the contact region (measurement points 1–19), the average surface hardness increase percentage is 8.29%, while at other locations beyond this region (measurement points 20–38), the average hardness growth rate is only 1.53%. Nevertheless, after enduring 100 freeze–thaw cycles, the average surface hardness of the model before the impact decreased by 18.9% compared to the not frozen-thawed model, and the magnitude of surface hardness growth of the model becomes more pronounced, as shown in Fig. 22b. The highest surface hardness growth rate of the frozen-thawed model is observed at detection point 3, with a value of 16.74%. Additionally, within the contact region, the average hardness growth rate is 12.53%. Therefore, both the maximum growth rate and the average growth rate of surface hardness within the contact region are higher in the frozen-thawed model compared to the not frozen-thawed model. This phenomenon implies that the influence of freeze–thaw cycles on the surface hardness of the track slab model primarily involves two aspects: on the one hand, after undergoing freeze–thaw cycles, the surface hardness of the model before impact noticeably decreases. On the other hand, after the ice block impact on the frozen-thawed model, the growth rate of surface hardness will increase by 51.45% compared to the not frozen-thawed model.

The decrease in surface hardness of the track slab model prior to impact, following freeze–thaw cycles, can be attributed to the repetitive freezing and thawing of water within the concrete pores. The physical mechanism responsible for freeze–thaw damage primarily involves water phase transition. When water freezes, it undergoes a volume expansion of approximately 9%, generating internal pressure within the concrete’s pores and cracks. If this pressure exceeds the tensile strength of concrete, new cracks will form or existing ones will propagate. Upon melting, water infiltrates deeper into the concrete, increasing the risk of further damage during subsequent freezing cycles. Cumulative damage occurs due to repeated freeze–thaw cycles, resulting in reduced strength and durability of the concrete through microcrack and fracture formation that diminish overall strength and density of the model. Additionally, these cycles disrupt cement paste-aggregate bonding leading to a decline in surface hardness.

However, when the ice block impacted the track slab model with energy from the impact, it caused further changes in the model. The impact induced local deformation and densification, especially within the contact region, which led to an increase in the hardness of the model surface. This additional energy input from the impact resulted in further compaction and hardening of the track slab model. Nevertheless, under identical ice block mass and impact velocity conditions, the degree of surface hardening after impact primarily depends on the inherent condition of the model itself.

As previously mentioned, freeze–thaw cycles induce repetitive freezing and thawing of water within the internal pores of concrete, resulting in expansion and contraction that generate internal stresses. Over time, these stresses progressively weaken the structural integrity of the model, rendering it susceptible to vulnerability. In this scenario, when subjected to freeze–thaw cycles, the track slab model exhibits an increased susceptibility to localized compaction and deformation within the concrete in the contact region under the impact force of the ice block. Consequently, a more pronounced enhancement in hardness is observed in the contact region of the frozen-thawed model after impact compared to that of the not frozen-thawed model.

#### Wave velocity results of the track slabs

**Figure 23:**
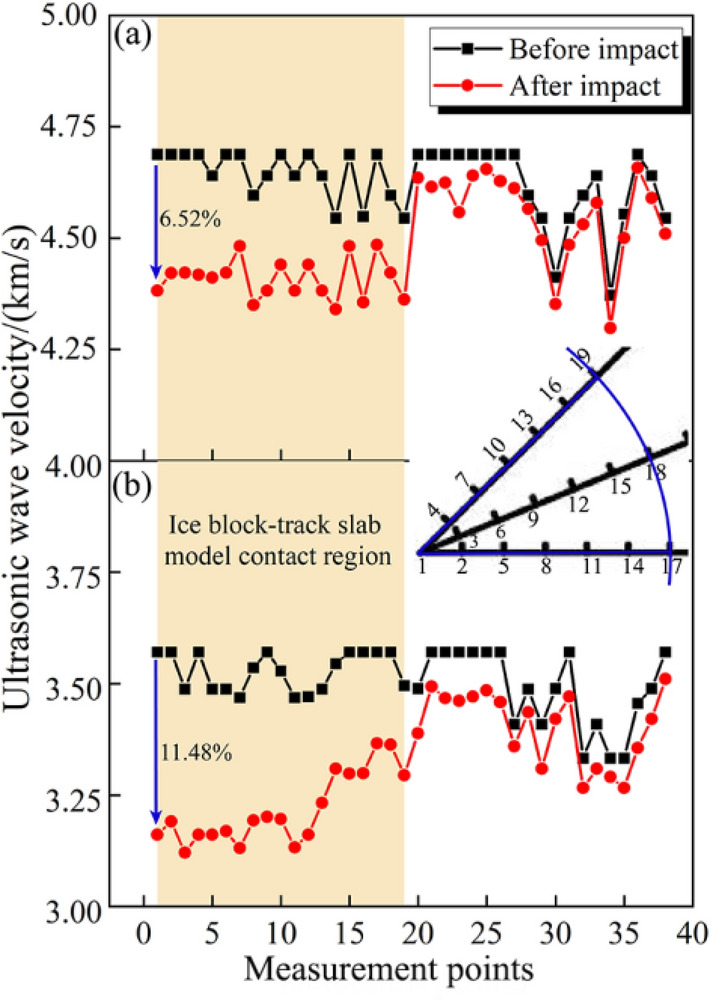
Ultrasonic wave velocity comparison before and after impact. (**a**) Not frozen-thawed model. (**b**) Frozen-thawed model.

The measurement data of wave velocity before and after impact are displayed in Fig. 23.

Figure 23a indicates that after the impact of the ice block, the wave velocity of the model will decrease apparently in the contact region. At measurement point 1, the wave velocity of the model exhibits the highest reduction rate, amounting to 6.52%. Moreover, within the contact region (measurement points 1–19), the wave velocity average decrease is significant, with a value of 5.08%. But at other measurement points beyond this region (measurement points 20–38), the average decrease is only 1.23%. However, after undergoing 100 freeze–thaw cycles, the average wave velocity of the model before the impact decreased by 24.25% compared to the not frozen-thawed model. Furthermore, there is a substantial increase in the rate of wave velocity decrease of the frozen-thawed model. Specifically, the location with the greatest wave velocity decrease is at measurement point 1, with a value of 11.48%. Within the contact region, the frozen-thawed model exhibits an average wave velocity decrease of 8.79%, while at other measurement points beyond this region, the average wave velocity decrease is only 2.35%. It is evident that the wave velocity decrease is pronounced in the contact region. Once outside this region, the decrease in wave velocity becomes less significant. Like the results of surface hardness, the influence of freeze–thaw cycles on the wave velocity of the track slab model also includes two aspects: firstly, the wave velocity of the frozen-thawed model significantly decreases before the impact, and secondly, the reduction rate of wave velocity in the frozen-thawed model increases by 73.03% compared to not frozen-thawed model after impact.

When the ice impacts the surface of the track slab model, it generates a stress wave that propagates through the concrete, inducing various forms of damage to its microstructure such as cracking, crushing, and fracturing. This leads to a reduction in both strength and stiffness. Moreover, when encountering boundaries like free surfaces, the stress wave undergoes reflection which results in complex stress fields within the contact zone. Such interference can exacerbate damage to the concrete structure, particularly within the regions that ice block and model contacted.

In general, the pores, cracks, and cumulative damage in the concrete act as obstacles for the sound wave, causing it to scatter, reflect, and attenuate. The sound wave loses energy as it travels through the damaged concrete, resulting in a decreased wave velocity. Consequently, this results in a more obvious decrease in wave velocity within the contact region. The decrease in wave velocity is less pronounced than the contact zone, as the stress wave attenuates while propagating away from the impact point. This attenuation is caused by energy dissipation due to material damping, geometric spreading, scattering, or mode conversion of the wave. The resulting attenuation leads to a reduction in both the intensity and damage potential of the stress wave, thereby resulting in reduced damage in areas beyond the contact region.

In summation, the combined utilization of the aforementioned three non-destructive testing methods not only allows for the effective localization of damage on the track slab model after being impacted by ice blocks but also provides specific numerical values of the dimple. This method enables a rapid, efficient, and straightforward preliminary assessment of track slab damage, laying the foundation for subsequent maintenance and repairs of the track slabs.

#### Preliminary quantitative damage evaluate of the track slab models

In this section, a preliminary evaluation of ice impact damage to the track slab model is conducted based on the aforementioned test results and in combination with existing concrete damage assessment methods.

As previously mentioned, a decrease in the velocity of ultrasonic waves typically indicates the presence of cracks or voids within the concrete, which impede wave propagation within the track slab model. Consequently, it becomes feasible to quantitatively evaluate the impact damage by calculating the rate of change in wave velocity. The rate of change in wave velocity can be determined using the following formula:

$$\Delta V=\frac{{V}_{0}-{V}_{d}}{{V}_{0}},$$

where $\Delta V$ represents the rate of change of wave velocity, ${V}_{0}$ is the wave velocity before impact, and ${V}_{d}$ is the wave velocity after impact. According to the rate of change in wave velocity, impact damage can be evaluated into three levels:

- *Minor damage* when the rate of change in wave velocity ($\Delta V$) is less than 5%, indicating the presence of microcracks on the surface of the track slab model with minimal influence on structural performance.
- *Moderate damage* when the rate of change in wave velocity ranges from 5 to 15%, suggesting an increased number of cracks within the track slab model that necessitate localized repairs.
- *Severe damage* when the rate of change in wave velocity exceeds 15%, signifying a significant decline in structural performance, potentially requiring comprehensive repair and reinforcement measures.

The change rate of surface hardness, similar to that of wave velocity, can also serve as an indicator for evaluating the impact damage of track slab models. The specific formula for calculating the rate of change in surface hardness is provided below:

$$\Delta H=\frac{{H}_{d}-{H}_{0}}{{H}_{0}}.$$

In formula, ∆*H* represents the rate of change of surface hardness, ${H}_{0}$ is the surface hardness before impact, and ${H}_{d}$ is the surface hardness after impact. According to the rate of change in surface hardness, impact damage can also be evaluated into three levels:

- *Minor damage* when the hardness change rate (∆*H*) Δ*H* < 10%, exhibiting negligible influence on structural performance.
- *Moderate damage* hardness change rate (∆*H*) 10% ≤ ∆*H* < 30%, resulting in a noticeable decrease in structural performance and evident surface damage.
- *Severe damage* hardness change rate ∆*H* ≥ 30%, leading to a significant deterioration in structural performance with extensive surface cracks, demanding immediate maintenance or reinforcement measures.

A preliminary assessment of damage was conducted on two types of track slab models. For the not frozen-thawed model, the contact region exhibited a 5.08% rate of change in wave velocity and an 8.29% rate of change in hardness. However, outside the contact region, there was only a 1.23% rate of change in wave velocity and a 1.53% rate of change in hardness. Consequently, based on the established method for evaluating damage, it can be determined that the not frozen-thawed model exhibited minor damage under ice impacts. Regarding the frozen thawed track slab model, within the contact region there was an 8.79% rate of change in wave velocity and a 12.53% rate of change in hardness observed; whereas outside this region there was only a 2.35% rate of change in wave velocity and a 2.31% rate of change in hardness recorded. Therefore, according to our evaluation method for assessing damage, it can be concluded that moderate damage occurred within the contact region for frozen-thawed track slab models while outside this region only minor damage was observed.

## Conclusions

A comprehensive analysis of the impact damage in the track slab model was performed by comparing its 3D surface morphology, surface hardness, and wave velocity before and after the impact test. Furthermore, differences in impact damage between frozen-thawed models and not frozen-thawed models were investigated. The main conclusions can be summarised as follows:

1. The test results confirm the feasibility and simplicity of the proposed non-destructive testing method, which can accurately assess the degree of track slab damage.
2. Upon being impacted by ice blocks, the track slab model undergoes irrecoverable deformation in the region of contact with the ice under the applied load, resulting in the formation of a small dimple on its surface with a diameter similar to that of the ice block. Furthermore, it is observed that this dimple forms near its central area.
3. After being impacted by the ice, the not frozen-thawed track slab model exhibits minor damage, and the maximum increase rate in surface hardness of the track slab model is 11.61%, while the maximum decrease rate in wave velocity is 6.52% within the contact region of the ice block and models. Outside this region, minimal changes are observed in both surface hardness and wave velocity.
4. After undergoing 100 freeze–thaw cycles, the model demonstrates moderate damage in the contact region, and exhibits a reduction in surface hardness and wave velocity prior to impact when compared to the not frozen-thawed model, with reductions of 18.9% and 24.25%, respectively. Furthermore, following an impact event with ice blocks, the maximum dimple depth increases by 35.74% compared to the not frozen-thawed model. The frozen-thawed model exhibits a maximum increase rate of 16.74% in surface hardness and a maximum decrease rate of 11.48% in wave velocity.

There are other problems worthy of future research efforts: such as, systematically investigate the mass and shape of ice, as well as the impact speed, angle, position, and frequency. Thoroughly explore the influence of loading parameters on the dynamic response of the track slab. Furthermore, it is essential to investigate how different forms of deteriorated track slab are affected by ice impacts and reveal the correlation mechanism and evolution pattern between impact parameters and damage characteristics. The content of this paper can be regarded as a preliminary study, which lays a solid foundation on providing mitigation strategies to reduce such impact damage in cold regions in the future.

## Data Availability

The data underlying this article will be shared on reasonable request to the corresponding author.
